# Understanding the role of digitalization and social media on energy citizenship

**DOI:** 10.12688/openreseurope.15267.1

**Published:** 2023-01-10

**Authors:** Lurian Klein, Ajesh Kumar, Annika Wolff, Bilal Naqvi

**Affiliations:** 1 Innovation Department, Cleanwatts Digital S.A., Ladeira da Paula 6, Coimbra, 3040-574, Portugal; 2 LUT School of Engineering Science, LUT University, Lappeenranta, Yliopistonkatu 34, 53850, Finland

**Keywords:** Digitalisation, social media, energy citizenship, energy informatics, energy literacy

## Abstract

The digitalisation of the energy domain can bring forth numerous aspects of the energy transition that can boost the emergence of energy citizenship, information sharing, and improved decision-making processes. However, this is premised on citizens being able to make sense of (digital) information. Hence, this paper proposes a link between energy informatics and energy citizenship via energy literacy, considering the cognitive and affective aspects of energy literacy and their relation to behaviour and action. By doing so, this paper aims to understand how the use of energy-related information and social media within five different case studies from the GRETA project can impel energy citizenship. This paper approaches this rationale through different means: (a) structured interviews to understand how citizens understand and make use of energy information within the case studies; (b) topic modelling on the content of those interviews to identify common factors that might spur on hinder behaviour change towards energy citizenship; and (c) social media content analysis to identify key energy-related topics of discussions among citizens around the globe and assess the role of social media as a tool for energy citizenship. As a result, this paper identified some key takeaways to improve the delivery of energy-related information to energy citizens for enhanced energy citizenship. These takeaways allow to conclude that it is fundamental to surpass the formal boundaries of techno-economic constructs and start addressing qualitative/subjective constructs (e.g., emotions, affections, and feelings) to foster energy citizenship. Also, these takeaways could be translated into social mechanism principles in the design of frontend energy-related digital platforms for improved end-user interactions and energy citizenship. Finally, this paper recognised the need to incentivise energy citizens to use social media for consuming energy-related information, and the need to formulate coordinated and coherent response strategies for disseminating energy-related information.

## 1 Introduction

The Horizon 2020 GRETA project ^1^ is funded under the LC-SC3-CC-1-2020 call focused on the “Social Sciences and Humanities aspects of the Clean Energy Transition” ^1^. Specifically, this call aims to shed light on cross-cutting issues impacting the clean energy transition beyond the technoeconomic realm, including for that matter socioeconomic, gender, sociocultural, and socio-political issues ^1^. One key question posed by this call is to better understand the impact that the digitisation of the energy system and social media have on the emergence and consolidation of energy citizenship ^1^.

The international treaties on climate change (e.g., the Paris Agreement) demonstrate the growing global awareness on the need to reduce greenhouse gas emissions and define pathways for a sustainable and citizen-centric energy transition - putting a strong emphasis on the adoption of renewable energy technologies and electric mobility and improving energy efficiency ^2^. At the same time, there has been an escalation of mobilizations by organized citizens, taking action to intervene in socio-political debates on climate change and the energy transition ^3^. Even though these manifestations are yet far from mainstream, their potential collective effects could be very significant for energy citizenship, which can be understood as the public’s awareness on their rights and responsibilities for collective energy actions and for a just, equitable, and sustainable energy transition ^4^. Therefore, energy citizenship is defined under the scope of the GRETA project as the notion of citizens as active, involved, and interested participants in the clean energy transition and the decarbonisation of energy systems, framed on the idea of society’s equitable rights and responsibilities to deal with the consequences of energy (mis)use – i.e., climate change. Contrarily to civic or social innovation initiatives that only offer incremental changes or innovative solutions to existing socio-technological systems, the GRETA project defends that energy citizenship has an inherent transformative nature that promotes real, radical changes in those systems.

On the other hand, digitalisation refers to the application of Information and Communication Technologies (ICT) across the economy to deliver new services, revenue streams, or value-producing opportunities – hence bridging the gap between the physical and the digital realms ^4^. The fast pace of digitalisation worldwide is only possible due to continuous advances in three main areas: Big Data, data analytics, and connectivity / interoperability ^5^. In the energy sector, digitalisation entails improved safety, productivity, efficiency, and sustainability of energy systems around the globe, as it facilitates the real-time management and operation of the grid ^5^. Illustratively, energy digitalisation can entail the use of real-time energy-related data to improve energy efficiency; or to develop models that improve customers’ experiences, information sharing, interactions, services, and decision-making processes.

Moreover, in terms of the impact of social media on energy citizenship, researchers have carried out experiments with energy citizens, energy data, and social media applications to see how it influences energy behaviour or usage pattern. Illustratively, from an experiment focused on the Facebook-based application, a significant reduction in energy consumption was observed in the case of socially enabled conditions ^6^. Social media was found to have enabled discussion around energy usage, an enhanced competition that resulted in motivation for energy savings, and socially mediated banter leading to an improved user experience ^6^. Researchers have also used other social media platforms like Twitter for studying how citizens are appropriating these platforms for their social and political needs ^6^. Furthermore, researchers have used sentiment and emotion analysis techniques on Twitter for understanding how the public discusses climate change and energy issues ^7^, and how to identify stroke survivors and their gender ^8^. Researchers have identified Twitter as a rich source for sentimental analysis and opinion mining on everyday aspect of life as well ^6^.

Hence, this paper defends the idea that the digitalisation of the energy domain and social media have the potential to bring forth numerous aspects of the energy transition that can boost the emergence of energy citizenship. However, this is premised on customers being able to make sense of such information.

In view of that, this paper proposes to look at how digitalisation and social media might impact the emergence and effectiveness of energy citizenship from an energy informatics and energy literacy perspective – i.e., how data and information derived from digitalisation and social media can support more educated energy-related decision-making processes and consequently spur energy citizenship.

To do so, this paper conducted a literature review on energy informatics and energy literacy to understand how the intersection between these two fields of research can potentially inform energy citizenship. Subsequently, this paper proposed three different methodological approaches to assess the impact that energy literacy and energy information deriving from digitisation and social media have on the emergence and consolidation of energy citizenship, including: (a) interviews with representatives from five GRETA case studies to understand how citizens currently understand and make use of energy information and social media to communicate about energy-related topics; (b) topic detection on the structured interview answers to detect common shortcomings and best practices related to the presentation and representation of energy information within the five GRETA case studies, as a means to provide inputs on the optimal design of energy-related digital platforms; and (c) social media content analysis to identify key energy-related topics of discussions among citizens to assess the role of social media as a tool for energy citizenship.

### 1.1 Case study description

The case studies under scrutiny in this paper are part of the Horizon 2020 GRETA project ^2^ – officially launched in 2021 and to be concluded in 2023. This Research & Innovation Action project aims to study the main determinants influencing the emergence of energy citizenship in different community-level contexts and scales, using for that matter a novel combination of scientific methods and models.

As previously explained, energy citizenship is defined under the scope of the GRETA project as a form of citizens’ proactive involvement and participation in the clean energy transition and the decarbonisation of energy systems in a fair and non-discriminatory manner, which can be manifested in multiple ways – e.g., households adopting renewable energy solutions or electric vehicles, citizens joining energy communities, or advocating for climate change, etc.

In view of that, the GRETA project aims to design and test different mechanisms of behavioural change capable of removing barriers to civic participation and empowerment in energy-related activities through enhanced energy citizenship (e.g., Energy Citizenship Contracts; Community Transition Pathways; Open Portfolio for Civic Energy Empowerment, etc.), as well as provide EU-level recommendations for improvements in the policy and legislative frameworks to include energy citizenship’s contribution towards decarbonisation goals.

**Figure 1.**
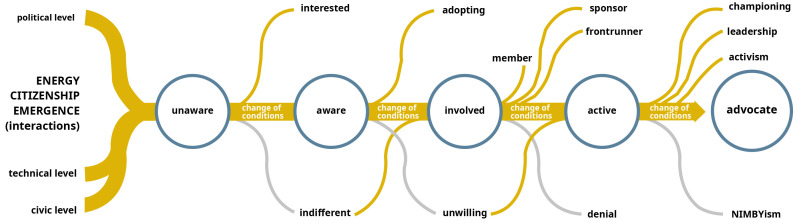
Representation of engagement transition outcomes along the GRETA's energy citizenship awareness ladder ^3^.

The GRETA project defends that civic empowerment is determined by a complex, cross-cutting mix of influencing factors that go far and beyond policy and regulation, including financial resources, access to technology, social dynamics, improved decision-making, and knowledge and information – and that changes in these factors might steer or hinder citizen motivations and willingness to engage in energy citizenship, thus positively or negatively impacting their level of engagement and agency. In view of that, the GRETA project conceptualised five different engagement transition levels along the GRETA energy citizenship awareness ladder, including: (i) unaware; (ii) aware; (iii) involved; (iv) active; (v) advocate. – as schematised in Figure 1.

To test that, the GRETA project conceptualised six case studies and an EU-wide multinational survey with citizens to identify and test what the determinants and levels that best impact the emergence of energy citizenship. On one hand, the six case studies serve to geographically anchor cases of emergence or inhibition of energy citizenship under different sociodemographic, socio-technological, socio-political (including institutional), governance, objectives, and geographical preconditions. On the other hand, the multinational survey with EU citizens (with approximately 10,000 responses) serves for standardization purposes – i.e., to correlate identified factors and levels of citizen involvement in a georeferenced way to test where and what type of energy citizenship is emerging. Table 1 provides an overview of the six GRETA case studies.

**Table 1.** Overview of the GRETA case studies.

**Case Study 1: Renewable Energy District - Bologna Pilastro-Roveri, Italy**			
**Geographical level**	**Governance**	**Main clean energy transition goal**	**Engagement level (pre and post GRETA)**
Local	Several associations / cooperatives	Renewable Energy	Unaware – Active
**Description**			
Mixed-use district in Bologna composed by two neighbourhoods: Pilastro (residential) and Roveri (industrial) and built as a response to the growing need of social housing for locating immigrants. Although Pilastro was conceived as an autonomous neighbourhood to be served by mixed services/activities, it became a mono-functional residential neighbourhood with socioeconomic issues (e.g., energy poverty). On the other hand, Roveri hosts a variety of companies in multiple sectors, and is involved in the creation of the first energy community of the city – benefiting from the presence of local industrial partners with large PV plants			
Case Study 2: Natural gas-free districts – The Netherlands			
Geographical level	Governance	Main clean energy transition goal	Engagement level (pre and post GRETA)
National	Cooperative / Municipalities	Renewable Energy / Energy Efficiency	Aware – Advocate
**Description**			
In 2019, the Dutch national program on natural gas-free neighbourhood initiated an initiative in the Netherlands for gas-free neighbourhoods by 2050 in view of increasing earthquakes near gas fields in Groningen. Municipalities are given the responsibility to ensure this sustainability transition. Need for citizen acceptance for investments on the renovation of the built environment towards natural gas-free heating infrastructures. Often the pilot projects are organised in the form of co-operative, who co-design this transition together with residents and with the techno-economic support from the municipality			
Case Study 3: Coopérnico - Renewable Energy Cooperative, Portugal			
Geographical level	Governance	Main clean energy transition goal	Engagement level (pre and post GRETA)
National	Cooperative	Renewable Energy / Energy Efficiency	Active - Advocate
**Description**			
Coopérnico is the first renewable energy cooperative in Portugal, counting with +1,000 members - including citizens, small-medium enterprises, and municipalities. Coopérnico’s mission is to involve its members to reshape the energy sector into a more renewable, socially just, and collaborative one. Coopérnico’s working areas are renewable electricity production, commercialisation, and innovative energy services – e.g., financing collective PV projects, supplying 100% renewable electricity to its clients, informing members about energy efficiency practices, supporting the creation of citizen energy communities, or lobbying at national level to support the citizens’ perspectives in the creation of energy policies and enabling legal frameworks			
Case Study 4: UR BEROA - Energy Efficiency Cooperative, Spain			
Geographical level	Governance	Main clean energy transition goal	Engagement level (pre and post GRETA)
Local	Cooperative	Energy Efficiency	Involved - Active
**Description**			
UR BEROA is a local energy efficiency cooperative in the neighbourhood of Bera Bera in San Sebastian. It is focused on delivering a sustainable plant composed of three natural gas boilers, a cogeneration engine, a boiler of biomass and solar panels that generate hot water – to be remotely managed based on the needs of each zone. UR BEROA is negotiating the offer of its services to a group of 237 households close to the neighbourhood, including collective self-consumption and other community-wide energy services / infrastructures (e.g., e-mobility)			
Case Study 5: Earnest App –Sustainability app for energy citizenship, Germany			
Geographical level	Governance	Main clean energy transition goal	Engagement level (pre and post GRETA)
Regional	Virtual community	E-Mobility	Aware - Active
**Description**			
The Earnest App is an is an informative, interactive and game-like online app that provides information and advice about energy and CO2 emissions. That is, it provides information, quizzes, and small challenges for its users to begin (or continue) to question their current energy and mobility behaviour (e.g., the use of e-mobility options, public transport, and reduction of long-term travel). It aims to encourage reflections on energy behaviour and – based on a growing understanding of the systemic consequences of mobility and consumption choices – seeks to incite spill over effects into adjacent areas of a sustainable lifestyle (e.g., energy efficiency). Case study members exchange their experiences with the app regularly online as part of a virtual community. Young citizens from the University of Applied Sciences in Darmstadt are the targeted demographics for the app.			
Case Study 6: Autonomous and connected electrical mobility network			
Geographical level	Governance	Main clean energy transition category goal	Engagement level (pre and post GRETA)
Supranational	Partnership	E-Mobility	Unaware - Active
**Description**			
The advent of electric autonomous mobility concerns the manufacture and integration of new electric vehicles, the upgrade of current road and traffic infrastructures, telecommunications based in 5G standards and battery recharging infrastructure. Assisted electric-autonomous mobility is likely to be widely available by 2025 and fully electric autonomous mobility by 2035. Roadmaps for pre-deployment and deployment of electric and autonomous mobility are in preparation within the European Cooperative, Connected and Automated Mobility (CCAM) Partnership - integrated by member state authorities, manufacturers, research and standardisation organisations, and the European Commission. The role of citizens in such initiative has been very limited, hence the rationales to study how the engagement of citizens is likely to emerge in this realm of sustainable energy transitions			

The different nature of the selected case studies was purposefully conceived to provide the GRETA project with a wide plethora of ways in which energy citizenship can best emerge.

It is important to note that the GRETA case study no. 5 (i.e., the Earnest App) was excluded from the scope of this paper as it went through an amendment process during the conduction of this study.

## 2 Literature review

### 2.1 Energy informatics and energy citizenship

Ryghaug *et al.* ^4^ argued that energy citizenship is based on material participation such as purchasing photovoltaic (PV) panels or using an Electric Vehicle (EV). The premise is that the act of owning and using such things reinforce energy citizenship. A criticism to this materialistic view is that it may exclude those who cannot afford the technology. Furthermore, in those cases people do not always interact with the technology directly, but instead the participation is mediated via data and other energy-related information. This might include, for example, looking at energy bills, monitoring usage through smart meters directly, or investigating the long-term cost and environmental benefits of owning PV panels, air-water heat pumps, EVs and so forth. As such, the sheer amount of data and other energy-related information might dissuade people from participating actively. To illustrate the inherent limitations of this materialistic view, a person might decide that it is not a good investment to install PV panel on their rooftop if the available area is insufficient or oriented in the wrong direction; if they are not at home during the day to make use of the generated energy; or if the cost of battery storage is prohibitive. However, this does not entail that they are not exercising energy citizenship, in case they have investigated the implications of such things, or if they use this knowledge to help others making decisions on whether to get PV panels – e.g., advocating to a neighbour who is at home during the day, who has a favourably oriented roof, or who may be eligible for a grant due to their socio-economic circumstance.

Therefore, this paper proposes to look at the intersection between energy information and energy citizenship from the perspective of non-material participation - e.g., how engaging with energy-related information may or may not support improved decision-making processes and consequently energy citizenship.

This paper fundaments this view from an energy informatics perspective, which is a field of research that is broadly concerned with how Information and Communication Technologies (ICT) is used within energy systems to capture high-level granular energy data for increased energy efficiency in energy distribution and consumption networks ^9, 10^. It has been seen that providing end-users with energy data and information does influence their energy consumption behaviours, which however rely on other societal factors and social dynamics ^11^. Hence, with the increasing availability of sophisticated IOT-based smart devices in households and adaptability of software applications in handheld devices, alongside the increasing accessibility to renewable generation, energy storage and electric mobility, research on energy informatics must start embracing a citizen-centric orientation to start promoting the optimisation of energy systems based on principles of energy justice and equity ^9^. In this regard, research on what kind of energy-related information is relevant to spur energy citizenship and actions among energy citizens has been identified as a strategic approach achieve that ^9^.

While some aspects of energy informatics concern cyber-physical systems (i.e., automated systems that use real-time data to drive automatic responses for improved energy efficiency), this paper is mostly concerned with the role of energy informatics in human decision-making. Hence, by taking the online definition of informatics ^12^ as:

*“Informatics harnesses the power and possibility of digital technology to transform data and information into knowledge that people use every day.”*

Then this paper proposes to similarly define energy informatics as:

*“Energy Informatics harnesses the power and possibility of digital technology to transform energy data and information into knowledge that people use every day.”*

Watson *et al.* ^9^ presented a research question matrix identifying nine important research questions concerning energy informatics, spanning different stakeholders - namely suppliers, consumers, and government. Some questions are more critical for energy citizenship than others, as they deal with the concept of information that can aid citizens to be more critically informed about their energy usage, including:

- How information systems can increase energy efficiency by integrating supply and demand data
- How information systems can be used to change social norms around energy consumption
- What data could aid in determining the efficiency of energy policy
- What information can help energy citizens to understand their own energy consumption through devices that they own

These are all important questions to look at when it comes to citizens and how they interact with the energy realm. These authors concluded that energy informatics can help increase energy efficiency, since the collection and analysis of energy-related data support the optimization of energy flows in the grid, and support citizens to better understand their energy behaviour. Similarly, Dao *et al.* ^13^ argued that a higher level of energy-related data granularity supports the development of information systems that optimise energy usage and aid sustainability. Moreover, Wilhite ^14^ and Yim ^11^ have shown that when information on energy consumption is provided (e.g., through energy bills or smart meters), it is effective in changing energy consumption behaviour. It has also been seen that when it comes to collective culture or community-wise energy projects, energy informatics has a significant role in increasing participation and engagement in energy conservation efforts, especially in communities where a strong collective culture exists ^11^.

### 2.2 Energy literacy and energy citizenship

Energy literacy, very broadly, refers to the ability to answer questions and solve energy-related problems. According to the U.S. Department of Energy ^15^, who have created an energy literacy framework to support teaching and learning about energy, an energy literate person: (i) can trace energy flows and think in terms of energy systems; (ii) knows how much energy they use, for what purpose, and where the energy comes from; (iii) can assess the credibility of information about energy; (iv) can communicate about energy and energy use in meaningful ways; (v) can make informed energy use decisions based on the understanding of its impacts and consequences.

Energy literacy is thought of comprising three main dimensions, including: (i) the cognitive dimension associated with the energy-related knowledge and skills abovementioned; (ii) the affective dimension associated with attitudes, values, and personal responsibility towards energy; and finally (ii) the behavioural dimension associated with the intention, involvement, and actions to be taken ^16^. Although these dimensions interact in complex ways, DeWaters and Powers ^17^ identified that the behavioural and affective dimensions may be more closely related with each other than with knowledge. As Martins *et al.* ^16^ also point out, most studies have shown that, in an overall, the general population has quite low levels of energy literacy and that negatively impacts their commitment towards energy saving behaviours. It is therefore clear that energy literacy plays a significant role in spurring energy citizenship.

Illustratively, Comeau *et al.* ^18^ explored this link in a Canadian context through a national survey undertaken by 3,000 citizens. The survey analysis found a high level of awareness and support for renewable energy among those citizens, and for improving energy efficiency in their home, but a general reluctance to take positive actions such as allowing utility suppliers to lower home or water temperatures remotely, or to install PV panels. The authors speculated that this could be due to a lack of detailed familiarity with these specific technologies ^18^. Similarly, they identified that Canadians were aware of ways that they could get involved in energy-related projects but failed to turn up to meetings due to several factors: lack of knowledge, a feeling that their participation was tokenistic, and a lack of trust in the organisations involved ^18^. Finally, they identified that most respondents favoured responsible energy use to protect the environment ^18^.

Resolving such issues require a multitude of approaches. In the GRETA project, the approach undertaken was trying to reduce barriers to support citizens engaging with energy information, so that the information derived from energy-related data could better inform and support decision-making processes or other energy-related behaviours. This more directly supports aspects of energy literacy related to the cognitive dimension proposed by Martins *et al.* ^16^, although it also relates to the affective and behavioural dimension as well.

**Figure 2.**
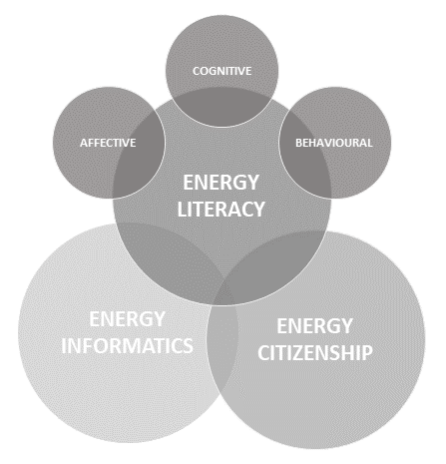
Relationship between energy informatics, energy citizenship, and energy literacy.

Based on the abovementioned, the relationship between energy informatics, energy citizenship, and energy literacy is drawn in Figure 2.

## 3 Methods

This section aims at presenting the distinct methodological approaches tailored in this study to assess the potential impact of digitalisation and social media (from an energy literacy and energy informatics perspective) on spurring energy citizenship – based on the assimilation of the main take-aways from the scientific literature. Firstly, this section describes the interview structure conducted with representatives from five GRETA case studies to understand how its citizens currently understand and make use of energy information and social media to communicate about energy-related topics. A topic detection exercise was performed on the interviews to detect common shortcomings and best practices related to the current ways of presenting and representing energy information within the five GRETA case studies, as to provide inputs on the optimal design of energy-related digital platforms. Additionally, based on insights from the interview answers, a broad social media content analysis exercise was conducted beyond the scope of the five GRETA case studies to identify key energy-related topics of discussion among citizens worldwide to assess the role of social media as a tool for energy citizenship.

### 3.1 Structured interviews

In line with the objectives set by this study, structured interviews with the five GRETA case studies were conducted to understand how they currently understand and make use of information and social media to communicate about energy-related topics with stakeholders – including energy citizens and the broad public. The structured interviews served to guide the development of two different methodologies to uncover the impact of digitalisation and social media on the emergence and consolidation of energy citizenship – i.e., the topic detection exercise and the social media content analysis.

Interviewing was the scientific approach elected by this study to gather data from the five GRETA case studies as it allowed researchers to: (i) access perception; (ii) understand how the respondent has constructed or developed meaning; and (iii) access the respondent’s version of the situation or reality as seen or experienced by them. A structured interview involves asking a set of predefined questions, thus allowing the standardisation of questions for each respondent and the comparison between different responses in a more structured manner ^19^.

Guidelines for structured interviews suggest that the interview must not be too long ^20^. Also, the scientific literature suggests that there must be a good rapport between the interviewer and the interviewed, which may be a challenge at times. The interview question should gradually increase in complexity and should be able to gather data relevant to the objective under perspective. This also requires that both the interviewer and the interviewed have relevant knowledge about the topic ^20^.

Following those guidelines, the interview protocol proposed in this study was conducted with representatives of five case studies within the GRETA project ^4^. These representatives were partners of the GRETA project consortium responsible for managing their respective case studies, as they held updated information about the day-to-day activities within the case studies and interacted with energy citizens regularly – thus representing gatekeepers capable of aligning the views and bridging the interactions between the project consortium and the five GRETA case studies.

The GRETA case studies are composed by many different stakeholders (e.g., governments who define energy policy frameworks, business entities focused on renewable energy, local municipalities who support the decarbonisation goals of neighbourhoods, and citizens who participate in the renewable energy transition, etc.). However, since the main target stakeholder group of the interviews were energy citizens, not all the energy information available within the GRETA case studies are relevant to them – e.g., technical information about transmission and distribution grids, to name one example. This was a relevant consideration for the creation of the structured interview protocol.

Additionally, it was identified that the types of available energy-related information within five GRETA case studies were twofold – i.e., energy-related information relevant across case studies or unique to each case study. Hence, following the scientific recommendations abovementioned and the specificities of the five GRETA case studies, the structured interview protocol aimed at seeking the following energy-related information: (i) why the renewable energy transition is important (e.g., the impact of reducing the carbon footprint, sustainable living, energy efficiency measures, renewable energy, etc. in climate change and global warming); (ii) what actions can be taken to achieve decarbonisation goals (e.g., step-by-step action guide for the renewable energy transition; relevant economic and social considerations; etc.); and (iii) how citizens can get involved (e.g., access to incentives or subsidies; improved and more educated decision-making; etc.).

Based on the abovementioned, a set of seven structured interviews were designed as presented in Table 2:

**Table 2.** List of interview questions.

No.	Question statement
1	In your own words, describe your case study, its community, and its main goal
2	What information is being presented to energy citizens in your case study?
3	What channels are being used for disseminating energy information – e.g., energy bills, mobile text messages, social media, website, booklets, etc.?
4	In your opinion, what role digitalisation [including use of (i) social media; (ii) energy-related digital platforms] can have in disseminating energy-related information among energy citizens? Does your case study have any presence on social media? If yes, what is the primary use for social media in your case study?
5	In your case study, how does the energy information is delivered among energy citizens? Actively: i.e., information is sent automatically by the systems based on usage and other parameters without the intermediation of the user. If so, what is the frequency? Passively/On Demand: i.e., the user must request information every time.
6	In your opinion, did the energy information presented to users ever changed their energy behaviour? If so, can you give examples of those changes? Did you ever spot differences in energy usage patterns from the cases where information is actively delivered versus passively/on demand?
7	Did energy citizens ever report any gaps (in terms of content, or presentation/visualisation of energy information)? In your opinion, how should energy information be ideally presented?

As the interviewees were situated in different geographical locations, video interviews were conducted online to take advantage of the synchronous mode of communication, which is considered a better mode of communication for the interview as it allows the participants to take advantage of non-verbal communication – which is also called social cues such as voice, intonation, and body language ^21^. Specifically, the interviews were conducted online through Microsoft Teams during the first week of November 2021, with an average duration of 50 minutes.

### 3.2 Topic detection on the structured interviews

This subsection aims to analyse the existing viewpoints on the current ways of presentation and representation of energy information among five GRETA case studies to distil common take-aways that could be translated into the design of energy-related digital platforms. The core idea behind this exercise is to give visibility to the potential shortcomings and good practices related to the presentation and representation of energy information in the GRETA project.

For that, the interview answers to questions no. 4-5-6-7 represented a fruitful textual source of evidence about the existing good practices and gaps on the presentation and representation of energy information among five GRETA case studies. Therefore, a rigorous text classification was performed on these open-ended answers, using a method entitled topic detection (also known as topic modelling or topic analysis) ^22^, following a similar methodological approach performed by Klein *et al.* ^23^. Through this method, it was possible to break down, extract, and categorise the most relevant parts-of-speech tags or key phrases from textual data into topics that summarise its core ideas, giving a complete picture of the topics discussed in a text corpus.

Furthermore, considering that the dataset contained in the interview questions no. 4-5-6-7 was not overly extensive, the topic detection was carried out manually with a proper level of accuracy and efficiency, which allowed to derive meaning and reveal patterns across the interviewed multiple viewpoints on the current way of presentation and representation of energy information in GRETA. This approach is in line with the criteria set by Podger *et al.* ^24^ with regards to the design of context-appropriate assessment methods and tools, which includes: (i) methodological rigour, richness, and reliability of results; (ii) adaptability to the target respondents and project specificities; (iii) ease of use resources for replicability.

### 3.3 Social media content analysis

Social media might represent an important tool for disseminating energy-related information and spurring energy citizenship, as the scientific literature points out that energy-related information shared in a social setting can lead to energy savings ^25^. However, its use is generally limited to a younger stratification of society and many individuals do not use it due to generational gaps ^26^.

In the context of the five GRETA case studies, it was identified that not all of them use social media as a regular channel for dissemination of energy-related information, and that social media might be seen as a potential source of misinformation spreading. In view of this limitation, this study decided to broaden the scope of the social media content analysis and target Twitter users across the globe rather than focusing on the five GRETA case studies.

Twitter was selected among different social media channels as it is widely used for such analysis since it allows the classification of content based on hashtags that generally represent the topic under discussion ^5^.

Specifically, a proprietary software entitled “Hashtagfy.me” was used to analyse Twitter trends for energy-related hashtags ^5^. This tool was selected from many other similar online tools as it allows the analysis of the engagement and popularity of a given topic through hashtag search as well as the measurement of the volume of discussion around a given topic. Hashtags were used because they collect different content under the same given topic based on the hashtag used to search for it. This helps to consider all the tweets that were posted online using the same hashtag or its slight variations. For instance, #cleanenergy, #renewableenergy, #solarenergy, #greenenergy, #sustainablity, and #energytransition represent the hashtags analysed using the proposed online tool, based on relevant topics discussed by the interviewees in subsection 3.1. English was the mainstream language for the search of hashtags as it was the predominant language used during the structured interviews presented in subsection 3.1.

## 4 Results

### 4.2 Topic detection on the structured interviews

Following the methodological approach described in subsection 3.2, the content of the open-ended interview answers to questions no. 4-5-6-7 was categorised into seven different gap-oriented topics (i.e., things that should be improved) and eight good practice-oriented topics (i.e., things that are currently done well). The correlation between these topics and their corresponding answers are presented in Table 3, together with the frequency in which these topics appeared across the interviewed answers.

**Table 3.** Topic detection exercise among five GRETA case studies.

Topic detection	Gap-oriented topics (i.e., what should be improved) [Case Study – CS] [Question – Q]	Frequency
**Ease-of-use, intuitive presentation /** ** visualisation of energy information**	CS1 - Q4: “Energy Monitoring platform could go a long way to communicate and visualise the information” CS2 - Q4: “Energy information could also help in visualisation of energy information” CS3 - Q7: “(…) they can't take advantage of the information presented – they don't know how to work with it, and they don't have interest despite being an easy way to obtain data” CS6 - Q5: “Platforms exist but less aware, consume time, need to know something about technology”	80%
**Simplification of complex / overly** ** technical information**	CS1 - Q7: “(…) easily explaining the benefit of the result to individual”; Q4: “If energy information systems are made easy to understand and use, they are useful” CS2 - Q4: “Less of technical and more easily understandable content” CS3 - Q7: “Information is not always easy to comprehend, that is, even if you try to simplify the content, there are situations in which you really have to talk to people” CS4 - Q6: “Information needs to be very clear, and information should be simplified”; “Consumer don’t understand technical information of energy bill”	80%
**Reduce overload of information**	CS3 - Q7: “(…) a lot of information in one document, e.g., invoices. They must have so much information that people sometimes give up trying to understand” CS6 - Q4: “As citizens use fully automated vehicle, people they will have more time, they will have mobile and internet, and it will create more data. Could create many services for user and risk and opportunities for manufacturer”	40%
**Inclusive, non-discriminatory** ** information sharing**	CS2 - Q4: “Energy information systems would be useful depending upon the message they convey and the kind of demographic they are catering to” CS4 - Q4: “(…) generational gap due to old age, thus communication strategy is still old fashion”	40%
**Awareness raising**	CS6 - Q7: “Media shows sense of urgency, but it does not translate to immediate consumption changes. Rebound effect over consumption. More awareness”	20%
**Retain attention**	CS3 - Q7: “(…) the problem here is after the activation phase (within the engagement process) where there is loss of interest in the platform (after 2 months it's not interesting anymore)”	20%
**Catering of trustworthy and** ** scientifically valid information**	CS2 - Q7: “Some problems municipality officials face due to social media disinformation as residents believe in wrong information at times. Municipalities need to step in shoes of citizens and cater to information that they need to make transition plan a success”	20%
**Good practice-oriented topics (how it should ideally be done) [Case Study – CS] [Question – Q]**		
**Humanisation of processes / social** ** value generation**	CS2 - Q6: “What we have seen in case studies that have been successful in involving people from start or involving people the way they feel comfortable with, is making it possible to have discussion with homeowners and talk about their worries and needs”; Q6: “How to be involved and feeling of being included in process (This is more important, that people have feeling of being included and be a part)”; “Trust is important, technology is secondary” CS3 - Q6: “Members had doubts about the indexed tariff and therefore Coopérnico needed to explain what consumption profiles, tariff components, etc. were. Example of Catarina as a member: the 1st time she felt the need for more information, she called and there was another person on the other end of the line (staff, not volunteer). She even found this strange (i.e., positively impacted) for not being a standard call centre call. Basically the modus operandi is members working for members”	40%
**Use of graphs / images**	CS3 - Q7: “(…) images or graphics are much easier to understand – e.g., the newsletter”	20%
**Digitalisation / dematerialisation of ** **information and processes**	CS4 - Q4: “Room for improvement on digitisation”; “Digitisation of relationship with member including billing and communication”	20%
**Use of metrics to present information**	CS1 - Q7: “Metrics that could denote consumption and benefits at individual and social level”	20%
**Create step-by-step guidelines**	CS1 - Q7: “Clear information on concrete steps to take”	20%
**Create new communication streams**	CS4 - Q4: “New communication strategy and technology needs to be further adopted”	20%
**Associate processes with a monetary ** **value**	CS1 - Q7: “Information about monetisation of the effort that would require individual to best behave towards energy”	20%
**Personalisation of the presentation of ** **information**	CS2 - Q4: “As people move up the steps in customer journey, the questions change from more general to more specific”	20%

In the same line of thought proposed by Klein *et al.* ^23^, it is important to note that the topic detection exercise was interpretative and adopted a social constructionist perspective. Hence, the interpretations made cannot be understood as definitive given that the proposed approach deals with uncertainty, multiple perspectives, and the absence of a single, universally valid answer to the topic detection exercise – i.e., they should be open to multiple rounds of reinterpretation.

The analysis of Table 3 revealed answers that were clearly articulated, multifaceted, and rich in details and complexity, often with a strong view on the existing shortcomings in each case study, as well as a pragmatic view on how things should be ideally done in a best-case scenario. This is evidenced in the examples showcased below:

*“Most people do not care about heat transition from natural gas to electric one, thus you must tailor the information and link it to things which people care about, to be able to get them on board.”*

*“(…) Basically (Coopérnico’s) modus operandi is members working for members.”*

This indicates that the interviewees could make sense of a complex social dimension, suggesting a strong commitment, knowledge, and real interest in their respective case study. Nonetheless, given that the topic exercise was solely based on the interviewed persons’ self-reported data, and in view to maintain a certain degree of “reflectiveness” about the proposed evaluation, it is essential to note that the interview responses might present some degree of Hawthorne effect – which stands for the idea that individuals (i.e., the interviewed) might modify some aspects of their behaviour (i.e., their answers) due to their awareness of being observed (i.e., interviewed) ^27^.

Furthermore, some topics stood out from others as they represented shared gaps and good practices across multiple case studies. The main common gaps uncovered by the topic detection exercise were: (i) the need for ease-of-use, intuitive presentation/visualisation of energy information; and (ii) the need for simplification of complex or overly technical information, which was present in 80% of the interviews (i.e., in four out of five interviews). On the other hand, the main common good practice uncovered by the topic detection exercise was the humanisation of processes / spurring social value generation, which was present in 40% of the interviews (i.e., in two out of five interviews). The uncovered topics that were transversal to different case studies are exceptionally valuable given that each interview was conducted individually (and without any connection to the other interviews) as to avoid conformity bias. Hence, a common thread binding different case studies was detected, even though they highly differ from one another (in terms of scales, geographic locations, legal frameworks, energy community configurations, sociodemographic characteristics, sociotechnical infrastructures, climate zones, etc.).

Based on the above analysis, it can be assumed that the interviewees proposed some valid gap-oriented and good practice-oriented recommendations that could be translated into the proposal of embedded social mechanisms in the design of energy-related digital platforms. On that topic, Annala *et al.* ^27^ described some core scientific fundaments from the SSH that informed the development of optimal user-centric frontend interfaces for end-user interaction in local energy markets, including: the provision of added value; promotion of capacity building and awareness raising; creation of commitment and appeal; ease-of- use and intuitive designs; incentivisation of social comparison; stimulation of reflection and learning; consideration of a community-wide perspective for problem solving; gamification approaches, among others.

By analysing the abovementioned information against the foundational core constructs raised from the SSH literature, and by comparing it with the gap- and good practice- topics identified in the topic detection exercise, it becomes clear that in order to strive and succeed in the optimal design of energy-related digital platforms, it is fundamental to surpass formal boundaries of techno-economic constructs, and start also addressing qualitative, subjective constructs, such as emotions, affections, and feelings.

### 4.3 Social media content analysis

The analysis of relevant hashtags using the online tool “Hashtagfy.me” considered the last 8-week’ worth of tweets (which represent the maximum length of analysis permitted by the online tool). The 8-week period (October 12, 2021 – December 7, 2021) was chosen against a shorter period to overcome the challenges thrown by shorter periods which may coincide with social media campaigns that may include relative hashtags and skew the sample collected.

It is also worth noting that the online tool “Hashtagfy.me” analyses hashtags based on a popularity score. The popularity score is based on the velocity with which new tweets are posted on Twitter all over the world. The highest velocity for a hashtag gets it a popularity score of 100%, the second position is calculated relative to the first one, and so forth.

**Figure 3.**
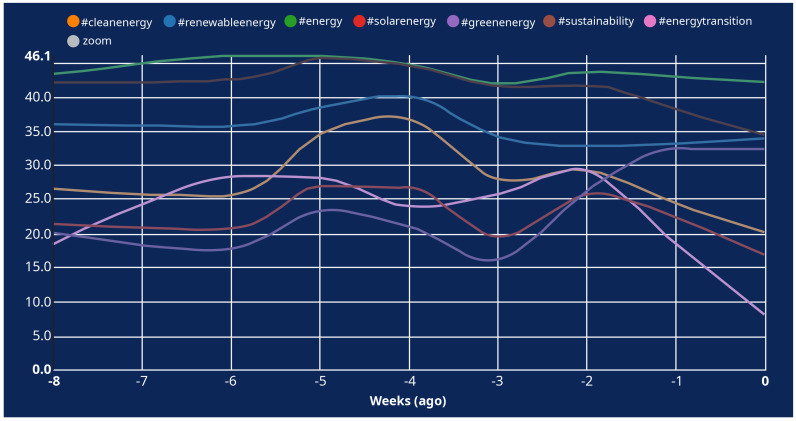
Popularity score of the selected energy-related hashtags on Twitter between Oct-Dec 2021.

**Figure 4.**
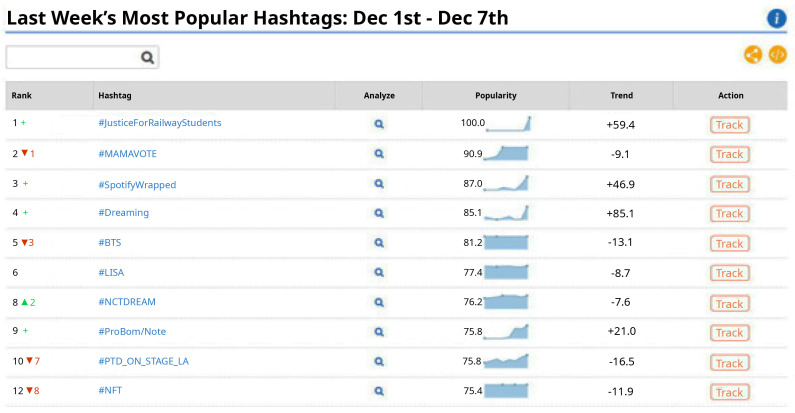
Popularity score of the topmost popular hashtags on Twitter between Oct-Dec 2021.

Figure 3 shows that there is a lot of variation in the popularity score of the selected energy-related hashtags, and that their popularity score is less than 50 in comparison to the most popular hashtags on Twitter during the 8-week analysis period (see Figure 4).

The online tool used in this study also revealed that the tweets posted on Twitter using the selected energy-related hashtags do not spur engagement among users as per the (absence of) likes and comments on them (except for a few exceptions – e.g., when the tweet is posted by an influential user, such as the celebrity Leonardo DiCaprio using the hashtags #cleanenergy and #energytransition).

Furthermore, in the case of the most popular hashtag on Twitter during the 8-week analysis period (i.e., #JusticeForRailwayStudents), its popularity score can be justified by a coordinated Twitter campaign ran by Indian students who demanded the Railway Recruitment Boards (i.e., a State-run transportation institution) new vacancies and a fair and timely recruitment process – making it the top trend with over three million tweets ^28^.

It can therefore be concluded that the role of social media (at least as pertaining to Twitter) on energy citizenship is yet to be exploited to its full potential, and it requires: (i) efforts to attract and incentivise different typologies of energy citizens to use social media for consuming and disseminating energy-related information and consequently spur energy citizenship; and (ii) formulating a coordinated and coherent response strategy for the dissemination of energy-related information.

## 5 Discussion and conclusion

This study aimed at understanding the impact of the digitalisation of energy systems and social media on the emergence and consolidation of energy citizenship, considering for this two intertwined perspectives: (i) energy informatics – which refers to the potential of ICT to transform energy data and energy-related information into knowledge that people use every day; and (ii) energy literacy – which refers to the ability to make sense of the energy data and energy-related information in improved decision-making processes. The broad literature research points out that energy literacy and energy informatics can positively impact sustainable energy-related behaviours, but there are still open questions about what information to be used and when, how it should be presented to different stakeholders, and how this relates to individual or collective energy literacies. To answer those open questions, three different methodologies were devised: (i) structured interviews with five GRETA case studies; (ii) topic detection on those interviews; (iii) and social media content analysis with a focus on Twitter.

The analysis of the structured interviews allowed to uncover three categories of information that are useful in engaging citizens in energy citizenship – i.e., information on why the energy transition is important, what actions can be taken to engage energy citizens in the decarbonisation of energy systems, and how citizens can get involved. The analysis of the structured interviews guided he development of two different methodologies to uncover the impact of digitalisation and social media on the emergence and consolidation of energy citizenship – i.e., the topic detection exercise and the social media content analysis.

Regarding the topic detection exercise on the structured interviews, different gaps and good practices on the current way that energy information is presented and represented among the five GRETA case studies were identified. The main gap-oriented topics uncovered were: (i) the need for ease-of-use, intuitive presentation/visualisation of energy information; and (ii) the need for simplification of complex/overly technical information. On the other hand, the main good practice-oriented topics uncovered was the humanisation of processes / spur social value generation. It is important to point out that these topics were transversal to different case studies – thus representing a common thread binding them regardless their contrasting configurations. These results showcase a potential effect on the affective dimensions of energy literacy, such as they relate to people’s attitudes and values. Not only that, but these gap-oriented and good practice-oriented topics could be translated into embedded social mechanisms in the design of frontend energy-related digital platforms for improved end-user interactions and energy citizenship. Hence, it can be concluded that the optimal design of energy-related digital platforms must start addressing qualitative, subjective constructs, such as emotions, affections, and feelings (i.e., the warming up of reason) in fostering energy citizenship, thus surpassing formal boundaries of techno-economic constructs.

Those reasonings align well with the proposed link made earlier between energy informatics and energy citizenship via energy literacy, since it considers not just the cognitive but also the affective aspects of energy literacy and their relation to energy informatics and energy citizenship and action. However, in view of time constraints, a caveat of this exercise relates to the fact that interviews were conducted with case study representatives (i.e., GRETA’s consortium partners) that offered insights on the end-users’ perspectives of their respective case studies, rather than directly with end-users per se (i.e., GRETA’s external stakeholders). Hence, other gaps and good practices than those highlighted in this study might had been uncovered in case end-users from each case study were the primary subject of the topic detection exercise.

Finally, with regards to social media (at least as pertaining to Twitter) as a tool for energy citizenship, it was identified that in addition to concerns about it potentially spreading misinformation, its impact on energy-related topics is not as strong as it could be. Hence, for it to be exploited to its full potential, it still requires: (i) efforts on the part of all stakeholders to use social media for disseminating energy information that aids towards energy citizenship; and (ii) the formulation of a coordinated and coherent response strategy for dissemination of energy-related content. However, the limitations presented by this method relate to the fact that it performed a global analysis of the Twitter hashtags, hence particular inferences about the five case studies were not made. Also, other social media were not considered in this study.

## Ethics and consent

Ethical approval was not required for this study, as the interviewees were partners of the GRETA consortium responsible for managing the case studies rather than external stakeholders per se (i.e., case study participants). As per the Grant Agreement No. 1011022317, the GRETA project activities include stakeholder engagement, data management, and result dissemination that is conducted in accordance with pertinent national and European regulations as well as Horizon 2020 recommendations and guidelines related to ethics and responsible research and innovation. The project partners involved with end-users are well trained and have considerable expertise in dealing with research involving people. Moreover, the project Executive Committee (E-COM) is responsible for guaranteeing that all the consortium members abide to lawful and ethical procedures during the development of the project. Verbal consent was obtained, and this was due to the participants being part of the GRETA consortium, which is in line with European regulations as well as Horizon 2020 recommendations and guidelines related to ethics and responsible research and innovation.

## Data Availability

As per the Grant Agreement No. 101022317, the GRETA project participates in the Open Research Data Pilot (ORDP) initiative and provides open access to research data and scientific publications whenever possible. Moreover, the Zenodo repository is used to ensure the maximum dissemination of the generated information within the project. However, the GRETA Data Management Plan (Deliverable D8.5) lists the following data sharing restrictions: (i) access to data and reports will be limited to members of the GRETA project until publications and presentations are submitted; (ii) some data and reports may be held back temporarily until the results are reviewed and validated by the appropriate intellectual property office at Lappeenranta-Lahti University of Technology (LUT) (GRETA’s project coordinator) or the partner institutions; (iii) some data might not be made available if deemed sensitive personal information by the supervising committee; (iv) proprietary information (if any) provided by commercial firms under a confidentiality agreement will not be made available without the consent and approval of the firms (Landeck J. A., 2021). Hence, in view of sensitive personal data concerns, the transcript of the interview data will not be shared publicly. If readers of this article wish to review the raw data, please contact the corresponding author Lurian Klein ( lklein@cleanwatts.energy). Access to this data is subject to approval and may be granted only if strictly and adequately necessary for the replication of this study for which the data was processed.

## Footnotes

^1^ The GRETA project ( www.projectgreta.eu) has received funding from the European Union’s Horizon 2020 Research & Innovation programme under grant agreement No 101022317.

^2^ GRETA has received funding from the European Union’s Horizon 2020 Research and Innovation Action (RIA) programme under Grant Agreement no. 101022317.

^3^ Figure 1 has been reproduced with permission from the copyright holder Massari, M. (2020) ( m.massari@unibo.it).

^4^ Although the GRETA project comprises six case studies, only five were involved in the study presented in this paper as one of them underwent changes at the time the interviews were conducted.

^5^ Freely available, open-source alternatives to the proprietary software used in this study include Tinfoleak ( https://github.com/vaguileradiaz/tinfoleak) or TAGSExplorer ( https://hawksey.info/tagsexplorer/), to name a few.
