# Supplementary material for: Understanding the role of digitalization and social media on energy citizenship
Source: Open Res Eur. 2023 Jan 10;3:6. [Version 1] doi: 10.12688/openreseurope.15267.1 (PMC10445890; doi:10.12688/openreseurope.15267.1)
Supplement: Peer review history

# Peer review: Reviewer response for version 1

**DOI:** 10.21956/openreseurope.16509.r32339

**Published:** 2023-06-28

**Authors:** Antti Silvast

**Affiliations:** 1 Department of Technology, Management and Economics, Technical University of Denmark, Lyngby, Denmark

The paper “Understanding the role of digitalization and social media on energy citizenship” examines the link between energy informatics and energy citizenship and focuses on three issues: energy literacy, the cognitive and affective aspects of energy literacy, and the relation of these to behaviour and action. The topics chosen could not be more timely as energy transitions continue and digitalisation is a topic of several high-level policy programs, research and development projects, and expert discourse, including in the EU and internationally. 

 I wholly support the starting points of the manuscript: increased understanding is needed of how digital infrastructures shape energy citizenship and how the increasing availability of data and information about energy systems depends on citizen activities in turn. 

 There are several positive contributions from this research. It cites some of the current literature and contains a well-elaborated study design in qualitative interviewing and analysis of social media. Sufficient details of the methods employed are included, including how they were tailored to the data gathering and analysis needs. 

 However, I have two main concerns about this work and would like to discuss whether the definition of “digitalisation” and “energy citizenship” respectively are wide enough for the task at hand. 

 First, the paper understands digitalisation as “the application of Information and Communication Technologies (ICT) across the economy to deliver new services, revenue streams, or value-producing opportunities” (p.3). On the one way, the concern is hence with the increasing application of ICTs, but this concern is not particularly novel in organisations and is not exclusively used, as also new services and values are mentioned. But this distinction between adding more ICTs and whole new value propositions for energy services could be sharpened, as the results clearly attend to both - e.g. adding ICTs via smart meters on p. 7 vs. the wider design-related considerations on p.10.  

 Second, and perhaps more pertinently, the paper clearly links citizenship with activity, involvement, and interest, but in quite a particular manner. It defines energy citizens as “active, involved, and interested participants in the clean energy transition and the decarbonisation of energy systems” (p.3). This designation shapes the study design, the findings, and how they are interpreted. 

 There is some consideration of “misinformation” (p.9) but I feel that many more thoroughgoing issues are not attended to. Should opposing the energy transition still be counted as part of energy citizenship and when outside of it? What is the relationship between energy democracy and energy citizenship - and what about citizens that are not supportive of democratic structures? While many commentators directly link citizen engagement and energy citizenship positively, an explanatory model such as yours should focus ‘symmetrically’ on engagement as well as opposition. Empirical cases (see review in Silvast & Valkenburg, 2023 ^1^) show that both support and opposition usually appear: including protesting and petitioning against developers and public authorities. These relations between support and opposition are not static and fixed once and for all but alternate during energy projects. 

 I would be curious to hear more about how you might understand opposition, if at all, using the study design and materials and whether it came across in the findings.

If applicable, is the statistical analysis and its interpretation appropriate?

Not applicable

Is the study design appropriate and is the work technically sound?

Partly

Is the work clearly and accurately presented and does it engage with the current literature?

Partly

Are the conclusions drawn adequately supported by the results?

Yes

Are sufficient details of methods and analysis provided to allow replication by others?

Yes

Are all the source data and materials underlying the results available?

Yes

Reviewer Expertise:

Science and Technology Studies, smart energy systems, energy citizenship

I confirm that I have read this submission and believe that I have an appropriate level of expertise to confirm that it is of an acceptable scientific standard, however I have significant reservations, as outlined above.

## References

[1] : Energy citizenship: A critical perspective. *Energy Research & Social Science* .2023;98: 10.1016/j.erss.2023.102995 10.1016/j.erss.2023.102995

# Peer review: Reviewer response for version 1

**DOI:** 10.21956/openreseurope.16509.r32334

**Published:** 2023-06-26

**Authors:** Alexandra Revez

**Affiliations:** 1 University College Cork, Cork, County Cork, Ireland

The paper proposes a link between energy informatics and energy citizenship via energy literacy. The purpose is to understand how the use of energy-related information and social media can be channeled to promote energy citizenship. The paper draws on five different case-studies from the GRETA project and data collection includes structured interviews and social media content analysis.

 It considers the impact of the digitalization processes, namely the potential of ICT to transform energy data and energy related information and energy literacy to consolidate energy citizenship.

 The premise of the paper is interesting and relevant and would gain some attention in the fields of energy transitions, energy democracy and energy policy. I find particularly engaging and interesting the sharing of empirical evidence.  

 However, I feel the literature review would be more impactful if the authors provided a more critical reading of existing literature. This refers to indicating some of the known problems associated with social media and climate/energy communications. For instance, the fact that the mass media reproduction of images and the excessive and overwhelming rate at which information is consumed, replicated and shared online has undermined our ability to reach out to each other in meaningful ways. Also, how these circulations and their use and manipulation by several stakeholders can also spark subversion and caricature, which often feed into narratives of climate denial; see for instance.

- Luke (2015 ^1^).
- O’Neill (2020 ^2^).

I would recommend addressing this gap by including some more critical insights into barriers and problems associated with digitalization and use of social media to foster energy citizenship. Also, some consideration of the dominance of the knowledge-deficit approach to citizen engagement would be fruitful. This speaks of the problem of narrowing citizen engagement with energy to issues focused on lack of knowledge, capacity or motivation. This approach assumes that public disinterest or dissent is due to a deficit in public understanding, and largely dismisses alternative framings and held values. Your framework is perhaps a way to go beyond this narrow framing and it would be valuable to highlight this.

**See work by:** Chilvers and Pallett (2018 ^3^).

**In terms of Research Design, the paper appears well structured** and grounded on engaging and relevant information. I also think the conclusion section could develop some recommendations for further research in light of these findings and the limitations outlined.

 Some additional minor points:

- The 1 ^st^ paragraph in the Introduction section that is giving a brief description of the GRETA project is in my opinion more suitably placed in the Case-study description section below.

If applicable, is the statistical analysis and its interpretation appropriate?

Yes

Is the study design appropriate and is the work technically sound?

Yes

Is the work clearly and accurately presented and does it engage with the current literature?

Partly

Are the conclusions drawn adequately supported by the results?

Yes

Are sufficient details of methods and analysis provided to allow replication by others?

Yes

Are all the source data and materials underlying the results available?

Yes

Reviewer Expertise:

Energy citizenship, energy democracy

I confirm that I have read this submission and believe that I have an appropriate level of expertise to confirm that it is of an acceptable scientific standard.

## References

[1] : The climate change imaginary. *Current Sociology* .2015;63(2) : 10.1177/0011392114556593 280-296 10.1177/0011392114556593

[2] : More than meets the eye: a longitudinal analysis of climate change imagery in the print media. *Climatic Change* .2020;163(1) : 10.1007/s10584-019-02504-8 9-26 10.1007/s10584-019-02504-8

[3] : Energy Democracies and Publics in the Making: A Relational Agenda for Research and Practice. *Frontiers in Communication* .2018;3: 10.3389/fcomm.2018.00014 10.3389/fcomm.2018.00014

# Peer review: Reviewer response for version 1

**DOI:** 10.21956/openreseurope.16509.r32602

**Published:** 2023-06-26

**Authors:** Dasom Lee

**Affiliations:** 1 Graduate School of Science and Technology Policy, Korea Advanced Institute of Science and Technology, Daejeon, Daejeon, South Korea

This paper argues that there is a link between energy informatics and energy citizenship via energy literacy. It uses several different methods: structured interviews, topic modeling, and social media content analysis. This paper has some fundamental problems such as the lack of research question and/or hypotheses, and it will need to be substantially revised in order for it to be indexed.

**1. Introduction:**

 2 ^nd^ paragraph: I disagree with the authors’ definition of energy citizenship. Energy citizenship is much broader than dealing with climate change, but it is the idea of having citizen led, publicly discussed energy systems that leads to a more democratic process.

 3 ^rd^ paragraph: I again disagree with the author’s definition of digitalization. Digitalization does not just focus on economy revenue, and value-producing. It is a socio-political and technological process.

 4 ^th^ paragraph: the authors need a better justification as to why they are focusing on social media when there are other means that leads to energy citizenship that has been suggested to be much more powerful such as community meetings and civil society organizations. Twitter might be a rich source for sentimental analysis, but is it a good enough source for energy citizenship? What kind of studies have used Twitter to show its link with energy citizenship? References are lacking and the discussions carried out in this paragraph are not in depth.

 5 ^th^ paragraph: Based on my comments above, I do not see enough evidence to suggest that the energy domain and social media are strongly linked. This is not to say that I am not open to the idea, I just need better formed arguments with more references.

**1.1. Case study description:**

 I really like Figure 1. I think it is great that the authors are developing such a theory. Based on Figure 1, it would be helpful if the authors can identify when energy citizenship starts – does it start at the aware stage or the involved stage? The reason why I ask this is because studies have shown that energy citizenship tends to be locally based (e.g., people living close to wind turbines protesting against the noise, NYMBYism etc.). Then how would a platform as global as Twitter help with the analysis? Does mentioning energy justice on Twitter a form of energy citizenship (is this a form of the involved stage or the active stage?) or does energy citizenship specifically require some kind of actual action such as protesting (which would be the advocate stage, I assume)?

 I also ask the authors to justify why these case studies are chosen.

**2. Literature Review:**

 The authors do not propose a research question or hypotheses. This is a serious problem that must be addressed.

**3. Methods:**

 The authors do not justify why they have chosen such methods. Choosing structured interviews is quite rare – it pretty much resembles questionnaires but tend to take longer and be more expensive to do. Therefore, why did they decide to do structured interviews instead of semi-structured or even unstructured? Why is structured interview a good method for this paper?

 The authors write that they conducted three methods in their abstract: structured interviews, topic modeling, and social media content analysis. This is not quite correct – the authors have collected data using two different methods: structured interviews and social media. And then analyzed the data using topic modeling and content analysis.

 The authors need to justify why they decided to use topic modeling. The structured interview questions were quite open ended, and by choosing topic modeling, the authors are losing the richness of data. So, what are the authors getting from the 5 study sites that they are not getting from the Twitter data? Why did they choose to do topic modeling for structured interview data when so much more sample is already available from Twitter, and its data uses a similar text-based analysis – content analysis?

 The authors should also justify why they are using Twitter. I do not think referencing the IEA helps here. I did a quick search and the reference that the authors provide does not mention Twitter – so the authors are using an incorrect reference, which needs to be rectified. But other than that, the goal of the IEA report is different from an academic paper. The IEA report is to discuss new ideas without original empirical data and to give brief summaries. The authors are writing an original academic article.

 The authors also need to justify why they are using content analysis.

 The authors write that lots of people did not use social media in their 5 study sites, so the authors decided to use global Twitter data instead. This raises two questions: (1) if people are not using social media very much in those 5 study sites and because of the low number of social media use, the authors had to substantially increase the sample, then how valid is this study? (2) the authors conduct the structured interviews in the 5 study sites and conducts a content analysis for all Twitter users. What is the goal of this study (this question goes back to the lack of research question/hypotheses)? How do the authors view the generalizability of this study? I can see two options: (1) the authors can delete the social media section, which I don’t think adds too much to the analysis and use both content analysis and discourse analysis for the interview data or, (2) the authors can start with the Twitter data and use the five sites as case studies. Then we get the broader scope and the general trend as well as the richness of the text data from structured interviews.

**4. Findings:**

 The authors provide no descriptive statistics.

 The authors are also using discourse analysis (i.e., quoting interviews) in the findings section, which is not mentioned in the methods section.

 The authors need to label the y axis on Figure 3.

 I am wondering whether using an online tool to pull Twitter data and copying and pasting the tables and figures is good enough for an original academic paper. From what I can see, the merit of this paper is the richness of the interviews.

 The discussion in the findings section is not supported by the findings shown (the last paragraph of 4.3.).

**5. Discussion and conclusion:**

 Because of the lack of the research question and hypotheses, I do find this section rather confusing. Nevertheless, the section is missing two important parts: (1) the contribution of the paper, and (2) how the findings expand and add to the existing theories that are discussed in the literature review.

**Minor:**

 The findings section starts with 4.2., not 4.1.

If applicable, is the statistical analysis and its interpretation appropriate?

No

Is the study design appropriate and is the work technically sound?

No

Is the work clearly and accurately presented and does it engage with the current literature?

No

Are the conclusions drawn adequately supported by the results?

No

Are sufficient details of methods and analysis provided to allow replication by others?

No

Are all the source data and materials underlying the results available?

No

Reviewer Expertise:

Energy transition, STS, Cyber-physical systems, Sustainability

I confirm that I have read this submission and believe that I have an appropriate level of expertise to state that I do not consider it to be of an acceptable scientific standard, for reasons outlined above.

# Peer review: Reviewer response for version 1

**DOI:** 10.21956/openreseurope.16509.r32338

**Published:** 2023-06-26

**Authors:** Ines Campos

**Affiliations:** 1 Centre for Ecology and Environmental Change, Lisbon, Portugal

I enjoyed reading this article and find it a very relevant paper, with an original research topic, which can contribute to current research on energy citizenship, energy literacy, and energy informatics. I think however the article could be further improved and offer some suggestions to the authors. 

 First, in the introduction, the article refers in the first paragraph to a key question posed in the call that the project leading to this research/article responded to. I find it could be more relevant to instead frame from a perspective of current literature, the relevance of looking into the intersection of digitalisation and energy citizenship, and how there is still a gap in the literature, which would offer a stronger entry point/framing for the article.

 Next, in the introduction, energy citizenship is defined by citing the definition of the GRETA project (a reference should also be provided), yet this is likewise the definition of the article, so it could be stated that energy citizenship is defined in this article as" given definition" (including references that back up this definition, which could be also references of the GRETA project).

 Also, in this section, and related to the above comment, energy citizenship is presented by the authors as being 'transformative' - again it would be relevant to refer to published evidence that backs this statement - what is this transformative nature? What evidence in the literature backs this statement?

 The research question could be more explicitly presented in the introduction. As it is now, the article states (in the last three paragraphs of the introduction), that it will look at 'how digitalisation and social media might impact the emergence and effectiveness of energy citizenship from an energy informatics and energy literacy perspective – i.e., how data and information derived from digitalisation and social media can support more educated energy-related decision-making processes and consequently spur energy citizenship." - The last sentence seems to be the guiding research question for the article, I suggest this is explicitly stated. 

 In the case study description (section 1.1), I find the reference to a survey that was done in the context of GRETA project is of little relevance to this specific article.

 Instead, I would rather have a bit more information on the case studies, namely: why were the specific case studies selected for the purposes of this article's study? What were the criteria for their selection? How can they contribute to addressing the research question?  

 As it is now, the only sentence hinting at why the case studies are chosen is "The different nature of the selected case studies was purposefully conceived to provide the GRETA project with a wide plethora of ways in which energy citizenship can best emerge." - which is a bit vague and does not tell us much about why the case studies are relevant to the particular research question of the article. 

 Regarding section 2 (literature review), the review part on energy informatics is quite good. However, the bit on energy citizenship, which tells us about what the paper proposes to look at (i.e., the perspective of non-material participation) is based on a critique of the material participation approach of Ryghaugh *et al.* but does not in rigor provide a literature review on the topic. Also, Ryghaugh and colleagues draw on a practice theory approach, which understands practices as relational and embedded in materials but also competencies, therefore, it is not straightforward to argue that their approach would not consider that e.g. advising a neighbor on the installation of PV panels, would not be an energy citizenship practice. This part of the literature review section should be further improved with a more elaborated review of existing literature on energy citizenship, thus offering a more robust conceptual background for the study. 

 By comparison, the literature review part on energy informatics and energy literacy is very good.

 In this section, the meaning of the following sentence is unclear: "In this regard, research on what kind of energy-related information is relevant to spur energy citizenship and actions among energy citizens has been identified as a strategic approach to achieve that".

 There are some minor English language and typo issues here and there, so the article should be carefully revised in this scope. 

 Concerning the methods, section 3, I have also a few minor notes:

 How many interviews were conducted? It is written that interviews were conducted with the five case studies - are these five interviews?

 Topic modeling usually refers to the integration of computational tools when performing topic detection as it implies a modeling/use of an algorithm and a logic to it - so it would be more accurate to simply refer to topic detection (the abstract refers to topic modeling). 

 The results are clear (would only review the use of the English language here). 

 The discussion and conclusion section is also clear, however, it could be further streamlined to specifically address the research question driving the article, which could further strengthen the conclusions. 

 Overall, I find the article very interesting and relevant and hope the suggestions provided help to further improve this work.

If applicable, is the statistical analysis and its interpretation appropriate?

Not applicable

Is the study design appropriate and is the work technically sound?

Yes

Is the work clearly and accurately presented and does it engage with the current literature?

Yes

Are the conclusions drawn adequately supported by the results?

Yes

Are sufficient details of methods and analysis provided to allow replication by others?

Partly

Are all the source data and materials underlying the results available?

Yes

Reviewer Expertise:

Social Sciences Sustainability and Energy Research

I confirm that I have read this submission and believe that I have an appropriate level of expertise to confirm that it is of an acceptable scientific standard, however I have significant reservations, as outlined above.
